# Post-replicative nick translation occurs on the lagging strand during prolonged depletion of DNA ligase I in *Saccharomyces cerevisiae*

**DOI:** 10.1093/g3journal/jkab205

**Published:** 2021-06-14

**Authors:** Natasha C Koussa, Duncan J Smith

**Affiliations:** Department of Biology, New York University, New York, NY 10003, USA

**Keywords:** DNA replication, DNA polymerase delta, DNA ligase I, lagging-strand synthesis

## Abstract

During lagging-strand synthesis, strand-displacement synthesis by DNA polymerase delta (Pol ∂), coupled to nucleolytic cleavage of DNA flap structures, produces a nick-translation reaction that replaces the DNA at the 5′ end of the preceding Okazaki fragment. Previous work following depletion of DNA ligase I in *Saccharomyces cerevisae* suggests that DNA-bound proteins, principally nucleosomes and the transcription factors Abf1/Rap1/Reb1, pose a barrier to Pol ∂ synthesis and thereby limit the extent of nick translation *in vivo*. However, the extended ligase depletion required for these experiments could lead to ongoing, non-physiological nick translation. Here, we investigate nick translation by analyzing Okazaki fragments purified after transient nuclear depletion of DNA ligase I in synchronized or asynchronous *Saccharomyces cerevisiae* cultures. We observe that, even with a short ligase depletion, Okazaki fragment termini are enriched around nucleosomes and Abf1/Reb1/Rap1-binding sites. However, protracted ligase depletion leads to a global change in the location of these termini, moving them toward nucleosome dyads from a more upstream location and further enriching termini at Abf1/Reb1/Rap1-binding sites. In addition, we observe an under-representation of DNA derived from DNA polymerase alpha—the polymerase that initiates Okazaki fragment synthesis—around the sites of Okazaki termini obtained from very brief ligase depletion. Our data suggest that, while nucleosomes and transcription factors do limit strand-displacement synthesis by Pol ∂ *in vivo*, post-replicative nick translation can occur at unligated Okazaki fragment termini such that previous analyses represent an overestimate of the extent of nick translation occurring during normal lagging-strand synthesis.

## Introduction

Half of all eukaryotic nuclear DNA is synthesized as Okazaki fragments on the lagging-strand. Synthesis of each Okazaki fragment requires several steps (reviewed in Balakrishnan and Bambara 2013; Burgers and Kunkel 2017): synthesis of an RNA primer by primase, addition of 10–35 DNA nucleotides to the 3′ end of this primer by DNA polymerase alpha (Pol α) (Nethanel and Kaufmann 1990; Perera et al. 2013), synthesis of the remainder of the Okazaki fragment by Pol ∂ (reviewed in Lujan, Williams, and Kunkel 2016; Guilliam and Yeeles 2020), and ultimately ligation by DNA ligase I—encoded by the *CDC9* gene in *Saccharomyces cerevisiae* (Johnston and Nasmyth 1978)—to generate a continuous strand. Prior to ligation, the RNA primer and at least some DNA from the 5′ end of the previously synthesized Okazaki fragment is removed via iterative cycles of strand-displacement synthesis and nucleolytic cleavage of the resulting flap structure (reviewed in Balakrishnan and Bambara 2013). The major Okazaki nuclease *in vivo* appears to be Fen1 (Rad27 in *S. cerevisiae*), with a minor contribution from Exo1 (Kahli et al. 2019): occasional excessive strand displacement in the absence of cleavage may generate long flap structures, which can be cleaved by Dna2 *in vitro* (Bae et al. 2001) and *in vivo* (Rossi et al. 2018). *In vitro*, Pol ∂ normally displaces only 1–2 nucleotides before Fen1 cleavage occurs (Stodola and Burgers 2016). Iterative rounds of strand-displacement synthesis and cleavage result in nick translation (Stith et al. 2008): alternatively, removal of the added nucleotide(s) by the 3′–5′ exonuclease activity of Pol ∂ can regenerate a ligatable nick via polymerase idling (Garg et al. 2004). 

Too little nick translation by Pol ∂ and its associated nucleases would leave DNA synthesized by the error-prone Pol α in the genome. However, excluding the seemingly unlikely scenario that Pol ∂ has a lower error rate or improved proofreading during strand-displacement synthesis, too much nick translation simply re-replicates DNA, hydrolyzing dNTPs without net synthesis. Excessive nick translation could also have negative effects on genome stability because Okazaki fragment ligation is required for the removal of PCNA from the nascent DNA by Elg1 (Kubota et al. 2015) and additionally occurs in competition with mismatch repair (Reyes et al. 2021). *In vitro*, the extent of nick translation by Pol δ/Fen1 inversely correlates with ligase concentration (Ayyagari et al. 2003). Moreover, if the 5′–3′ helicase Pif1 is included in such reactions, nick translation appears to predominate over ligation (Pike et al. 2009).

In *S. cerevisiae*, we have previously shown that the termini of Okazaki fragments enriched by extended DNA ligase I depletion are phased by nucleosomes and are also enriched upstream of binding sites for the transcription factors Abf1/Reb1/Rap1 (A/R/R) (Smith and Whitehouse 2012). These data suggest a model whereby proteins that immediately re-bind on the nascent lagging-strand constrain synthesis by Pol ∂, leading to an enrichment of ligation junctions at these obstacles. Consistent with this model, both nucleosomes (Devbhandari et al. 2017) and Reb1 (Sparks et al. 2020) impede Pol ∂ *in vitro*. We have also shown that extended co-depletion of both DNA ligase I and the major Okazaki nucleases shifts these termini upstream of the nucleosome dyad relative to extended ligase depletion alone (Kahli et al. 2019). *S. cerevisiae* lacks DNA ligase 3, which is able to serve as a backup ligase during Okazaki fragment biogenesis in multicellular eukaryotes (Arakawa et al. 2012; Kumamoto et al. 2021). Therefore, nicks on the lagging-strand in yeast cannot be sealed in the absence of DNA ligase I: the continued presence of PCNA-bound DNA nicks during prolonged ligase depletion may allow more extensive nick translation to occur under these conditions. Therefore, the extent to which nick translation occurs during normal, unperturbed Okazaki fragment biogenesis *in vivo* has been difficult to ascertain.

We and others (McGuffee et al. 2013; Petryk et al. 2016; Pourkarimi et al. 2016; Osmundson et al. 2017; Tubbs et al. 2018; Chen et al. 2019; Yeung and Smith 2020; Porcella et al. 2020; Koussa and Smith 2021) have previously used Okazaki fragment strand bias from asynchronous cultures to assay replication direction genome-wide, allowing quantitative analysis of replication initiation and termination. We were recently prompted to conduct an analysis of how apparent origin firing efficiency from these experiments varies through S-phase in synchronized cultures. We observed that apparent firing efficiency inferred from Okazaki fragment distributions evolves over S-phase, while remaining quantitatively accurate in the context of asynchronous populations with similar cell-cycle distributions (Koussa and Smith 2021). Prompted by this analysis, and in light of the more rapid DNA ligase depletion that can be achieved via anchor away (Haruki et al. 2008) relative to transcriptional repression or degrons, we sought to closely examine whether the position of Okazaki fragment termini remains stable over the course of replication.

By depleting DNA ligase I from the nucleus of *S. cerevisiae* cells for periods of 5–60 min, in either asynchronous or synchronized cell populations, we observe that extended depletion of DNA ligase results in a migration of Okazaki fragment termini toward nucleosome dyads and A/R/R sites from more upstream sites. We show that this effect cannot be explained by changes in Okazaki fragment processing at different stages of S-phase, suggesting that the prolonged absence of ligase leads to an increase in the extent of nick translation via continued nick translation. Finally, we compare previously reported maps of replicative DNA polymerase usage (Zhou et al. 2019) to the locations of Okazaki fragment termini obtained after very brief ligase depletion. We show a depletion of Pol α-derived DNA in the region immediately downstream of Okazaki fragment termini that have not been shifted by extensive nick translation and propose that these sites represent an accurate reflection of where Okazaki fragment ligation normally occurs *in vivo*.

## Materials and methods

### Yeast strains

**Figure 1:**
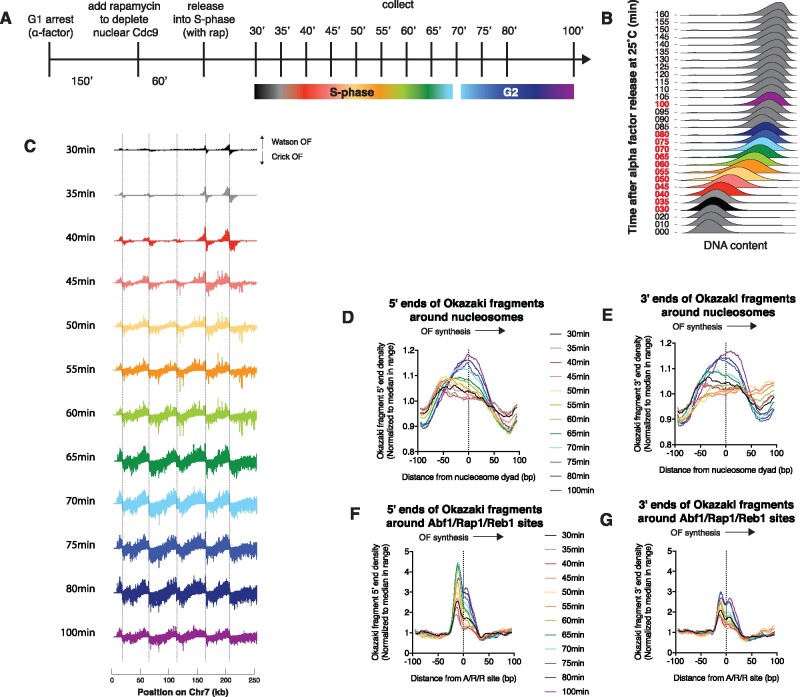
Okazaki fragment termini move during a synchronous S-phase in the absence of nuclear DNA ligase I. (A) Schematic representation of synchronous time course with continuous ligase depletion. (B) DNA content measured by flow cytometry. Red timepoints were sequenced. (C) Okazaki fragment sequencing data for the experiment shown in the figure across a portion of chromosome 7 for cultures treated with rapamycin to deplete Cdc9 from the nucleus by anchor away. Watson-strand fragments deriving from leftward-moving forks are shown above the axis, and Crick-strand fragments deriving from rightward forks are shown below the axis. Dashed lines represent known replication origins. Distribution of Okazaki fragment 5′ (D) and 3′ (E) ends around the midpoints of high-confidence nucleosomes (Jiang and Pugh 2009) at the indicated time after alpha-factor release in the presence of rapamycin. Data are smoothed to 5 bp and normalized to the median value in the range ±100 bp from the nucleosome midpoint. Distribution of Okazaki fragment 5′ (F) and 3′ (G) ends around Abf1/Reb1/Rap1 sites (MacIsaac et al. 2006) at the indicated time after alpha-factor release in the presence of rapamycin. Data are smoothed to 5 bp and normalized to the median value in the range ±100 bp from the transcription-factor-binding site midpoint.

All strains were derived from W303 RAD5+ background and contained additional mutations required for anchor-away depletion of Cdc9. The genotype of the wild-type strain is *tor1-1::HIS3*, *fpr1::NatMX4*, *RPL13A-2xFKBP12::TRP1*, and *CDC9-FRB::HygMX*. The experiment in Figure 1 uses a *POL3-AID* strain in which the Pol3 subunit of Pol ∂ is fused to an auxin-inducible degron as described in Koussa and Smith (2021). Pol ∂ levels in this strain are normal in the absence of 3-indoleacetic acid, and the strain therefore behaves as a wild type.

### Cell growth and cell-cycle synchronization

Yeast was grown in YPD at 30°C unless indicated otherwise. Rapamycin (Spectrum 41810000-2) was added at 1 μg/mL for the indicated time for Okazaki fragment analysis. For synchronized S-phase analysis, log phase cells (OD 0.2) were briefly sonicated to disrupt clumps and 10 μg/mL alpha factor was added, followed by 5 μg/mL every hour until >95% cells were fully shmooed. To release cells from arrest, cells were washed twice with deionized water and resuspended in YPD with or without rapamycin as required.

### Flow cytometry

Cells were collected by adding 150 μL of yeast culture to 350 μL absolute ethanol and stored at 4°C. Samples were treated with RNase by pelleting cells and resuspending in 500 μL of 50 mM sodium citrate with 42 μg/mL RNase A and incubating at 50°C for 2 h, followed by the addition of 100 μg proteinase K for two additional hours. Equal volume of 50 mM sodium citrate with 0.2 μL SYTOX green (Fisher S7020) was added, and samples were sonicated and analyzed on a Becton Dickinson Accuri or Cytek Aurora.

### Okazaki fragment preparation, labeling, and sequencing

Okazaki fragments were purified, end-labeled and deep-sequenced as previously described (Smith and Whitehouse 2012). Briefly, Okazaki fragments were purified from genomic DNA collected after Cdc9 depletion, using sequential elutions from Source 15Q columns. DNA was treated with RNase before adaptor ligation, second-strand synthesis, and barcoding. Paired-end sequencing (2 × 75 bp) was carried out on an Illumina Next-seq 500 platform.

### Computational analyses

FASTQ files were aligned to the S288C reference genome (SGD, R64-2-1) using the Bowtie (v2.2.9). Low-quality reads and PCR duplicates were removed and resulting data were converted to BEDPE files using the Samtools suite (v1.3.1). For Okazaki fragment sequencing, genome coverage was calculated using the Bedtools suite (v2.26.0) in a strand-specific manner. Origin efficiency metric analysis of predefined origins was carried out as previously described (McGuffee et al. 2013) with the origin list from the same source. Briefly, the fraction of Okazaki fragments mapping to the Watson or Crick strand in 10-kb windows to the left and right of the origin (W_L_, W_R_, C_L_, and C_R_) is calculated for each origin. OEM is calculated as W_L_/(W_L_ + C_L_) − W_R_/(W_R_ + C_R_). Okazaki fragment ends were aligned to consensus nucleosome dyads (Jiang and Pugh 2009) or Abf1/Reb1/Rap1 sites (MacIsaac et al. 2006) and normalized to the median end density in a 200-bp window around the sites. For HydEN-seq, reads from Zhou et al. (2019) were aligned to the genome as above and duplicates were removed. The location of ribonucleotides was determined as in Clausen et al. (2015). Okazaki fragment ends were aligned to consensus nucleosome dyads or Abf1/Reb1/Rap1 sites and normalized to the median end density in a 200-bp window around the sites as for Okazaki fragment analysis. Calculations were carried out in R with custom in-house scripts.

## Results

### Okazaki fragment termini move during a synchronous S-phase in the absence of nuclear DNA ligase I

We first analyzed whether the location of Okazaki fragment termini changed over the course of a synchronous S-phase in the absence of DNA ligase I. *CDC9-FRB* cells, in which the Cdc9 protein encoding DNA ligase I can be depleted from the nucleus via anchor away (Haruki et al. 2008), were arrested in G1 with alpha factor, treated with rapamycin for 1 h to deplete nuclear Cdc9, and released into a synchronous S-phase in the continued presence of rapamycin (Figure 1A). Cells were released at 25°C to slow replication and allow us to collect more timepoints during S-phase: cell-cycle analysis is shown in Figure 1B. Okazaki fragments from each timepoint were analyzed by paired-end sequencing to identify the location of the 5′ and 3′ termini. As expected, we observed that Okazaki fragments are enriched around efficient origins at early timepoints and appear more distal to origins as replication progresses (Figure 1C). Our sequencing libraries are prepared using a method that involves extensive RNase treatment and exposure to high pH, and which requires a 5′ phosphate on the DNA fragment for ligation to the upstream sequencing adaptor. Thus, we only sequence ligation-competent lagging-strand intermediates from which the RNA primer has been removed by nick translation.

Meta-analysis of Okazaki fragment termini around nucleosomes indicated that both the 5′ (Figure 1D) and 3′ (Figure 1E) termini of Okazaki fragments obtained at earlier timepoints were enriched upstream of nucleosome dyads, while those obtained at later timepoints were more symmetrically distributed around the nucleosome midpoint. In addition, the number of Okazaki fragment 5′ and 3′ termini found in the prominent peak at A/R/R sites increased as replication progresses (Figure 1, F and G). This change in the location of lagging-strand nicks could be explained by differences in synthesis, processing, or ligation at different stages of S-phase, or by ongoing lagging-strand processing at nicks remaining behind replication forks during the prolonged absence of ligase.

### The duration of ligase repression influences the location of Okazaki fragment termini in asynchronous cultures

**Figure 2:**
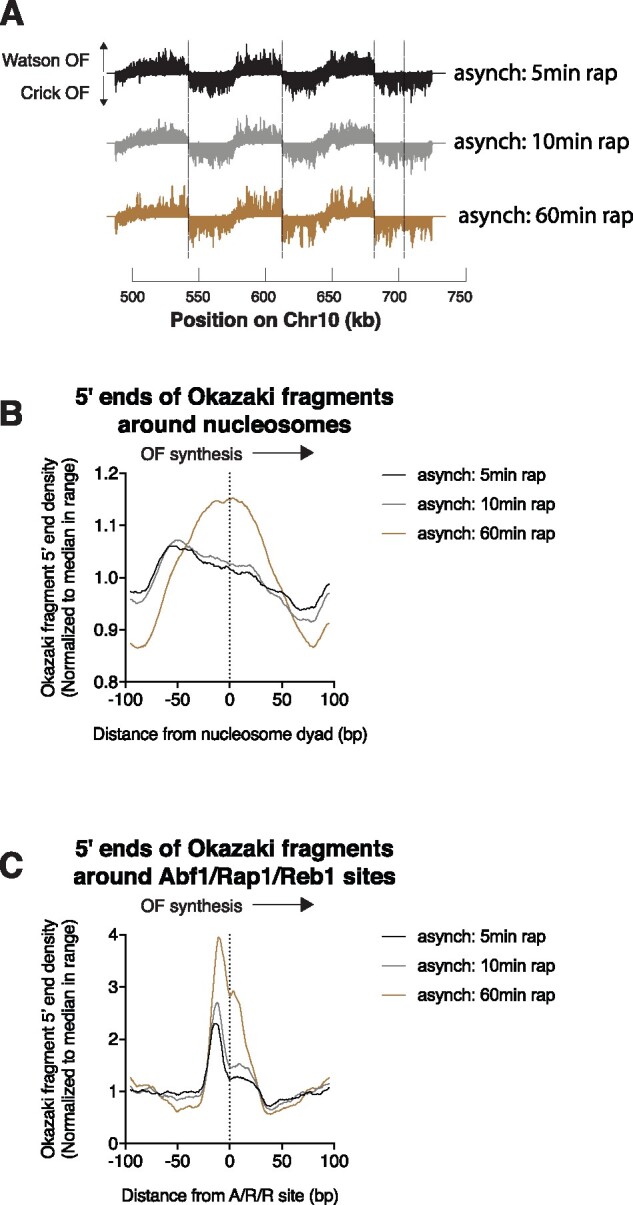
The duration of ligase repression influences the location of Okazaki fragment termini in asynchronous cultures. (A) Okazaki fragment sequencing data across the left arm of chromosome 10 for cultures treated with rapamycin for 5, 10, or 60 min, as in Fig. 1C. (B) Distribution of Okazaki fragment 5′ ends from synchronous cultures, treated with rapamycin as indicated, around nucleosomes. Data are presented as in Fig. 1D. (C) Distribution of Okazaki fragment 5′ ends from synchronous cultures, treated with rapamycin as indicated, around Abf1/Reb1/Rap1 sites. Data are presented as in Fig. 1F.

If ongoing Okazaki fragment processing occurs during protracted ligase depletion, Okazaki fragment termini obtained after very brief ligase depletion in asynchronous cells should be enriched upstream of nucleosome dyads compared to longer depletions. To test this, we analyzed Okazaki fragments from asynchronous cultures of *CDC9-FRB* cells treated with rapamycin for 5, 10, or 60 min. Okazaki fragments showed the anticipated strand bias even with this short depletion (Figure 2A), confirming that our sequencing data come from *bona fide* lagging-strand replication intermediates. As observed for timepoints early in S-phase with synchronous cultures, termini peaked upstream of the meta-nucleosome dyad after 5- or 10-min rapamycin treatment, while a 60-min depletion generated a roughly symmetrical distribution (Figure 2B). The difference between the 5- and 10-min timepoints was minimal, with at most a very slight shift in terminus location (Figure 2, B and C). We observed consistent results at A/R/R sites (Figure 2C). The height of the peak upstream of A/R/R sites was substantially higher after 60 min of ligase depletion than after 5 and increased only slightly in between the two early timepoints. These data are most consistent with ongoing lagging-strand processing occurring in the absence of DNA ligase activity.

### Okazaki fragment processing does not change through S-phase

**Figure 3:**
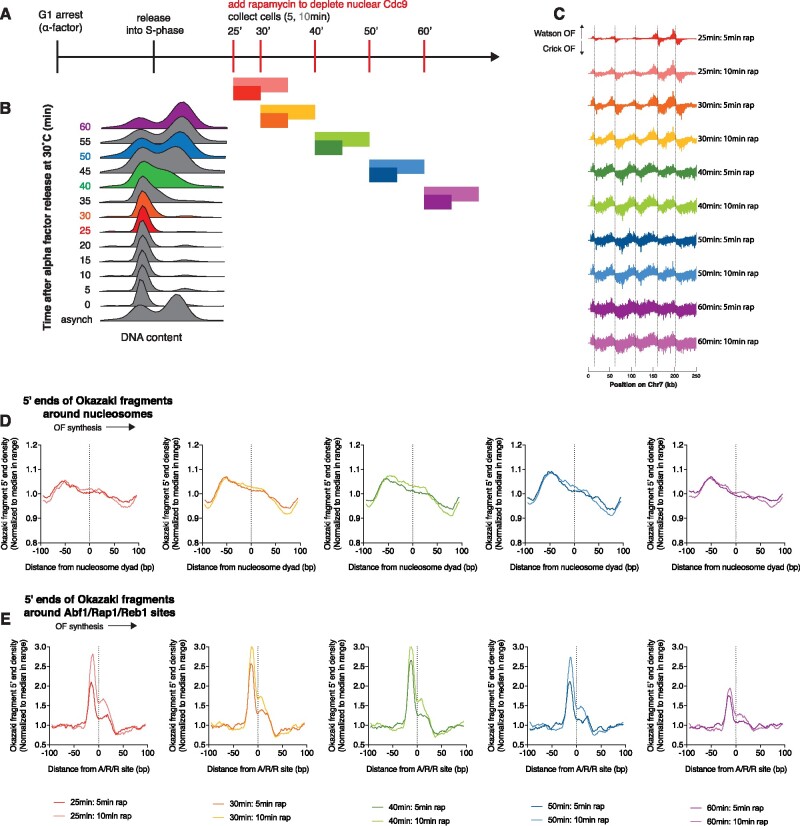
Okazaki fragment processing does not change through S-phase. (A) Schematic representation of synchronous brief ligase-depletion time course. (B) DNA content measured by flow cytometry for the samples shown in Fig. 2 and this figure. Red timepoints were sequenced. (C) Okazaki fragment sequencing data across a portion of chromosome 7 for synchronized cultures treated with rapamycin for 5 or 10 min to deplete Cdc9 from the nucleus by anchor away, as in Fig. 1C. (D) Distribution of Okazaki fragment 5′ ends around nucleosomes from cultures treated with rapamycin for 5 or 10 min at the indicated time after alpha-factor release. Data are presented as in Fig. 1D. (E) Distribution of Okazaki fragment 5′ ends around Abf1/Reb1/Rap1 sites from cultures treated with rapamycin for 5 or 10 min at the indicated time after alpha-factor release. Data are presented as in Fig. 1F.

The data shown in Figure 1 were obtained during an S-phase time course with continuous ligase depletion. As such, early timepoints analyze only Okazaki fragments that have just replicated, whereas timepoints from late S-phase report on Okazaki fragments from both early and late S-phase. To confirm that these data were not biased by differences in Okazaki fragment processing in early *vs* late S-phase, we analyzed Okazaki fragments from synchronous cultures subjected to transient, brief ligase depletion. Cells were released into S-phase, and at various points in S-phase cells were treated with rapamycin for either 5 or 10 min to deplete nuclear Cdc9 (Figure 3A). We sequenced samples to capture very early and very late S-phase, as well as intermediate timepoints (Figure 3B).

Okazaki fragments were enriched proximal to known origins at early timepoints and more distal to known origins at later timepoints (Figure 3C), confirming both that these are *bona fide* lagging-strand intermediates and that replication was synchronous. The genomic regions covered by sequencing reads after 10 min of treatment are also wider than after 5 min, indicating that substantially more replication has occurred during the 10-min treatment compared to the 5-min treatment. Importantly, the under-representation of Okazaki fragments proximal to origins at later timepoints indicates that early-replicating Okazaki fragments have already been ligated, rendering them immune to ligase depletion and invisible to our sequencing assay. Therefore, this analysis specifically interrogates Okazaki fragments synthesized during the period of rapamycin treatment.

At all timepoints analyzed (25, 30, 40, 50, and 60 min), the location of Okazaki fragment 5′ and 3′ termini obtained after 5 or 10 min of ligase repression was similar to the analogous distribution from an asynchronous culture (Figure 3D and Supplementary Figure S2A). Termini were enriched upstream of nucleosome dyads, with at most a minor shift between the two timepoints. As expected, and again similarly to data from asynchronous cultures (Figure 2), all timepoints also showed a peak of Okazaki fragment ends upstream of A/R/R sites (Figure 3E and Supplementary Figure S2B), and the height of the peak increased from the 5-min sample to the 10-min sample. The heights of these peaks, and overall shapes of the distributions, were very similar at all timepoints. Therefore, we conclude that the stage of S-phase does not significantly impact the location of Okazaki fragment termini in relation to protein barriers but that the duration of ligase depletion does. Together, the data in Figures 1–3 demonstrate that widespread ongoing nick translation occurs at lagging-strand nicks left unsealed by the prolonged absence of DNA ligase I.

### Comparison of Pol α-derived DNA to Okazaki fragment termini obtained from brief ligase depletion

**Figure 4:**
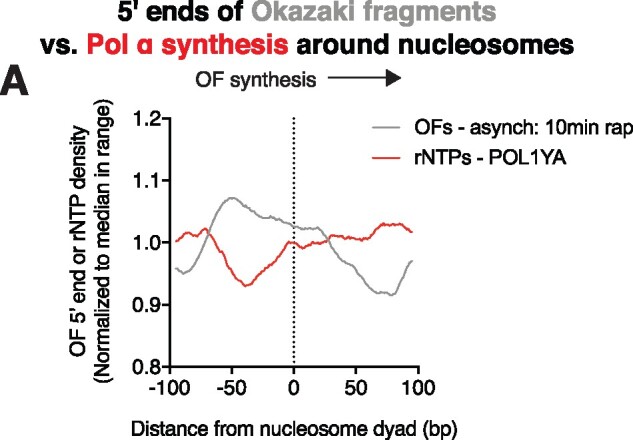
Comparison of Pol α-derived DNA to Okazaki fragment termini obtained from brief ligase depletion. (A) Distribution of Okazaki fragment 5′ ends around the midpoints of high-confidence nucleosomes (Jiang and Pugh 2009) in asynchronous cultures with 10 min of rapamycin (gray). Ribonucleotides from asynchronous cultures of POL1-Y869A rnh201Δ strain (red) are from (Zhou et al. 2019). Data are smoothed to 5 bp and normalized to the median value in the range ±100 bp from the nucleosome midpoint.

Our data suggest that, in cells proficient for DNA ligase, Okazaki fragment ligation normally occurs upstream of—as opposed to at—nucleosome dyads. Pol α is relatively error prone, and it has been proposed that retained Pol α-derived DNA is the cause of increased mutations observed near nucleosome dyads (Reijns et al. 2015). It is not clear how much of the Pol α-derived DNA primer is replaced by Pol δ synthesis. Polymerase use can be tracked mapping genomic ribonucleotides incorporated by a mutant allele of the relevant polymerase in a strain lacking RNase H2 (Koh et al. 2015; Clausen et al. 2015; Daigaku et al. 2015; Zhou et al. 2019). We compared the previously reported locations of Pol α-derived ribonucleotides incorporated by the *pol1-Y869A* allele (Zhou et al. 2019) to those of Okazaki fragment termini obtained after brief ligase depletion, and therefore not subject to additional nick translation (Figure 4). We observed a pronounced depletion of Pol α-derived DNA close to, but slightly downstream of, the peak of Okazaki fragment end density. We favor the interpretation that Pol α-derived DNA in this region is the most likely to be removed by Okazaki fragment processing. However, we cannot formally exclude two alternative possibilities—namely that the location upstream of the nucleosome dyad is disfavored for priming and that the *pol1-Y869A* mutation (Zhou et al. 2019) affects priming activity or location. Our favored interpretation implies that Okazaki fragments in ligase-proficient cells normally experience approximately as much strand displacement synthesis as we capture after a 5-min ligase repression.

## Discussion

The data presented here demonstrate that persistent nicks on the lagging-strand resulting from the prolonged absence of DNA ligase I are subject to ongoing strand-displacement synthesis and nucleolytic processing. This processing is likely facilitated by the PCNA that remains bound at such nicks in the absence of ligation (Kubota et al. 2015). We cannot distinguish between repeated binding and dissociation of Pol ∂ and Fen1 at these sites, or the continued presence of these proteins associated with PCNA. Transient ligase depletion generates a pattern of Okazaki fragment termini that more closely predicts the distribution of DNA synthesized by Pol αin *S. cerevisiae*; this suggests that our previously reported data on Okazaki fragment terminus location (Smith and Whitehouse 2012; Osmundson et al. 2017; Kahli et al. 2019) represent an overestimate of the true extent of nick translation *in vivo* due to their reliance on extended ligase depletion.

Although previous data likely overestimated the extent to which Pol ∂ carries out strand-displacement synthesis, brief ligase depletion suggests that the obstacles to Pol ∂ synthesis identified in this prior work—namely nucleosomes and select transcription factors—do represent true obstacles that impede Pol ∂ synthesis during lagging-strand synthesis. The observation that lagging-strand nicks become increasingly enriched around these obstacles during protracted nick translation suggests the same obstacles impede Pol ∂ whether nick translation is coupled to replication or not. Therefore, it appears that strand-displacement synthesis and nuclease cleavage are not fundamentally different whether they occur at the replication fork or on already-replicated DNA (*i.e.*, during repair synthesis). We note that Pol ∂ is impeded by protein obstacles in reconstituted systems that do not include other replisome components and that accessory helicases such as Pif1 reduce the extent of this impediment without eliminatinig it (Devbhandari et al. 2017; Sparks et al. 2020).

Although we observe an evolution in the location of Okazaki fragment termini as the duration of ligase depletion increases, the most significant change occurs between 60 and 70 min in cultures synchronously released into S-phase (Figure 1D), coincident with the end of S-phase (Figure 1B). We and others have reported that ligation of Okazaki fragments can be deferred to G2 (Kahli et al. 2019, Reyes et al. 2021), analogous to other repair processes. It is possible that strand displacement is somewhat more efficient in G2 than in S-phase, either due to globally increased repair or to decreased competition for necessary replication factors—for example, dNTPs, Pol ∂, or helicases.

Okazaki fragment sequencing (Ok-seq) remains a powerful method to investigate replication origin firing and replication termination genome-wide and has yielded important insights in *S. cerevisiae* (McGuffee et al. 2013; Osmundson et al. 2017), *C. elegans* (Pourkarimi et al. 2016) and mammalian cell lines (Petryk et al. 2016; Tubbs et al. 2018; Chen et al. 2019). The ability to capture sufficient Okazaki fragments to analyze replication-fork movement after only 5 min of ligase depletion will allow the analysis of *S. cerevisiae* strains for which more extended ligase depletion would be problematic, for example, mutants whose replication would be compromised or otherwise impacted by the accumulation of substantial numbers of DNA nicks.

## Data availability

Sequencing data have been submitted to the GEO under accession number GSE173065. Supplementary material available via figshare: https://doi.org/10.25387/g3.14535636.

## References

[jkab205-B1] Arakawa H , Bednar T, Wang M, Paul K, Mladenov E, et al 2012. Functional redundancy between DNA ligases i and III in DNA replication in vertebrate cells. Nucleic Acids Res. 40. doi:10.1093/nar/gkr1024. 10.1093/nar/gkr1024 PMC3315315 22127868

[jkab205-B2] Ayyagari R , Gomes XV, Gordenin DA, Burgers PMJ. 2003. Okazaki fragment maturation in yeast: I. Distribution of functions between FEN1 and DNA2. J Biol Chem. doi:10.1074/jbc.M209801200. 10.1074/jbc.M209801200 12424238

[jkab205-B3] Bae SH , Bae KH, Kim JA, Seo YS. 2001. RPA governs endonuclease switching during processing of Okazaki fragments in eukaryotes. Nature. 412:456–461. doi:10.1038/35086609. 11473323 10.1038/35086609

[jkab205-B4] Balakrishnan L , Bambara RA. 2013. Okazaki fragment metabolism. Cold Spring Harb Perspect Biol.5. doi:10.1101/cshperspect.a010173. 10.1101/cshperspect.a010173 PMC3552508 23378587

[jkab205-B5] Burgers PMJ , Kunkel TA. 2017. Eukaryotic DNA replication fork. Annu Rev Biochem. 86:417–438. doi:10.1146/annurev-biochem-061516-044709. 28301743 10.1146/annurev-biochem-061516-044709 PMC5597965

[jkab205-B6] Chen YH , Keegan S, Kahli M, Tonzi P, Fenyö D, et al 2019. Transcription shapes DNA replication initiation and termination in human cells. Nat Struct Mol Biol. 26: 67–77. doi:10.1038/s41594-018-0171-0. 30598550 10.1038/s41594-018-0171-0 PMC6320713

[jkab205-B7] Clausen AR , Lujan SA, Burkholder AB, Orebaugh CD, Williams JS, Clausen MF, et al 2015. Tracking replication enzymology in vivo by genome-wide mapping of ribonucleotide incorporation. Nat Struct Mol Biol. 22: 185–191. doi:10.1038/nsmb.2957. 25622295 10.1038/nsmb.2957 PMC4351163

[jkab205-B8] Daigaku Y , Keszthelyi A, Müller CA, Miyabe I, Brooks T, et al 2015. A global profile of replicative polymerase usage. Nat Struct Mol Biol. 22: 192–198. doi:10.1038/nsmb.2962. 25664722 10.1038/nsmb.2962 PMC4789492

[jkab205-B9] Devbhandari S , Jiang J, Kumar C, Whitehouse I, Remus D. 2017. Chromatin constrains the initiation and elongation of DNA replication. Mol Cell. doi:10.1016/j.molcel.2016.10.035. 10.1016/j.molcel.2016.10.035 PMC5256687 27989437

[jkab205-B10] Garg P , Stith CM, Sabouri N, Johansson E, Burgers PM. 2004. Idling by DNA polymerase δ maintains a ligatable nick during lagging-strand DNA replication. Genes Dev. doi:10.1101/gad.1252304. 10.1101/gad.1252304 PMC528896 15520275

[jkab205-B11] Guilliam TA , Yeeles JTP. 2020. An updated perspective on the polymerase division of labor during eukaryotic DNA replication. Crit Rev Biochem Mol Biol. doi:10.1080/10409238.2020.1811630. 10.1080/10409238.2020.1811630 32883112

[jkab205-B12] Haruki H , Nishikawa J, Laemmli UK. 2008. The anchor-away technique: rapid, conditional establishment of yeast mutant phenotypes. Mol Cell. 31:925–932. doi:10.1016/j.molcel.2008.07.020. 18922474 10.1016/j.molcel.2008.07.020

[jkab205-B13] Jiang C , Pugh BF. 2009. A compiled and systematic reference map of nucleosome positions across the Saccharomyces cerevisiae genome. Genome Biol. 10:r109.doi1186/gb-2009-10-10-r109. 19814794 10.1186/gb-2009-10-10-r109 PMC2784324

[jkab205-B14] Johnston LH , Nasmyth KA. 1978. Saccharomyces cerevisiae cell cycle mutant cdc9 is defective in DNA ligase. Nature. 274:891–893. doi:10.1038/274a0. 355897 10.1038/274891a0

[jkab205-B15] Kahli M , Osmundson JS, Yeung R, Smith DJ. 2019. Processing of eukaryotic Okazaki fragments by redundant nucleases can be uncoupled from ongoing DNA replication in vivo. Nucleic Acids Res. doi:10.1093/nar/gky1242. 10.1093/nar/gky1242 PMC6393292 30541106

[jkab205-B16] Koh KD , Balachander S, Hesselberth JR, Storici F. 2015. Ribose-seq: global mapping of ribonucleotides embedded in genomic DNA. Nat Methods. 12: 251–257. doi:10.1038/nmeth.3259. 25622106 10.1038/nmeth.3259 PMC4686381

[jkab205-B17] Koussa NC , Smith DJ. 2021. Limiting DNA polymerase delta alters replication dynamics and leads to a dependence on checkpoint activation and recombination-mediated DNA repair. PLoS Genet.17: e1009322.doi:10.1371/journal.pgen.1009322. 33493195 10.1371/journal.pgen.1009322 PMC7861531

[jkab205-B18] Kubota T , Katou Y, Nakato R, Shirahige K, Donaldson AD. 2015. Replication-coupled PCNA unloading by the Elg1 complex occurs genome-wide and requires Okazaki fragment ligation. Cell Rep. doi:10.1016/j.celrep.2015.06.066. 10.1016/j.celrep.2015.06.066 PMC4534484 26212319

[jkab205-B19] Kumamoto S , Nishiyama A, Chiba Y, Miyashita R, Konishi C, et al 2021. HPF1-dependent PARP activation promotes LIG3-XRCC1-mediated backup pathway of Okazaki fragment ligation. Nucleic Acids Res. 1–14. doi:10.1093/nar/gkab269. 33872376 10.1093/nar/gkab269 PMC8136790

[jkab205-B20] Lujan SA , Williams JS, Kunkel TA. 2016. DNA polymerases divide the labor of genome replication. Trends Cell Biol. 26. doi:10.1016/j.tcb.2016.04.012. 10.1016/j.tcb.2016.04.012 PMC4993630 27262731

[jkab205-B21] MacIsaac KD , Wang T, Gordon DB, Gifford DK, Stormo GD, et al 2006. An improved map of conserved regulatory sites for Saccharomyces cerevisiae. BMC Bioinformatics. 7:113.doi:10.1186/1471-2105. 16522208 10.1186/1471-2105-7-113 PMC1435934

[jkab205-B22] McGuffee SR , Smith DJ, Whitehouse I. 2013. Quantitative, genome-wide analysis of eukaryotic replication initiation and termination. Mol Cell. doi:10.1016/j.molcel.2013.03.004. 10.1016/j.molcel.2013.03.004 PMC3628276 23562327

[jkab205-B23] Nethanel T , Kaufmann G. 1990. Two DNA polymerases may be required for synthesis of the lagging DNA strand of simian virus 40. J Virol. 64: 5912–5918. doi:10.1128/jvi.64.12.1990. 2173773 10.1128/jvi.64.12.5912-5918.1990 PMC248760

[jkab205-B24] Osmundson JS , Kumar J, Yeung R, Smith DJ. 2017. Pif1-family helicases cooperatively suppress widespread replication-fork arrest at tRNA genes. Nat Struct Mol Biol. 162–170. 24:doi:10.1038/nsmb.3342. 27991904 10.1038/nsmb.3342 PMC5296403

[jkab205-B25] Perera RL , Torella R, Klinge S, Kilkenny ML, Maman JD, et al 2013. Mechanism for priming DNA synthesis by yeast DNA polymerase α. eLife. 2. doi:10.7554/eLife.00482. 10.7554/eLife.00482 PMC3628110 23599895

[jkab205-B26] Petryk N , Kahli M, d'Aubenton-Carafa Y, Jaszczyszyn Y, Shen Y, et al 2016. Replication landscape of the human genome. Nat Commun.7: 10208.doi:10.1038/ncomms10208. 26751768 10.1038/ncomms10208 PMC4729899

[jkab205-B27] Pike JE , Burgers PMJ, Campbell JL, Bambara RA. 2009. Pif1 helicase lengthens some Okazaki fragment flaps necessitating Dna2 nuclease/helicase action in the two-nuclease processing pathway. J Biol Chem. 284. doi:10.1074/jbc.M109.023325. 10.1074/jbc.M109.023325 PMC2757220 19605347

[jkab205-B28] Porcella SY , Koussa NC, Tang CP, Kramer DN, Srivastava P, et al 2020. Separable, Ctf4-mediated recruitment of DNA polymerase α for initiation of DNA synthesis at replication origins and lagging-strand priming during replication elongation. PLoS Genet.16: e1008755.doi:10.1371/journal.pgen.1008755. 32379761 10.1371/journal.pgen.1008755 PMC7237047

[jkab205-B29] Pourkarimi E , Bellush JM, Whitehouse I. 2016. Spatiotemporal coupling and decoupling of gene transcription with DNA replication origins during embryogenesis in *C. elegans*. eLife. 5. doi:10.7554/elife.21728. 10.7554/eLife.21728 PMC5222557 28009254

[jkab205-B30] Reijns MAM , Kemp H, Ding J, De Procé SM, Jackson AP, et al 2015. Lagging-strand replication shapes the mutational landscape of the genome. Nature. 518: 502–506. doi:10.1038/nature14183. 25624100 10.1038/nature14183 PMC4374164

[jkab205-B31] Reyes GX , Kolodziejczak A, Devakumar LJPS, Kubota T, Kolodner RD, et al 2021. Ligation of newly replicated DNA controls the timing of DNA mismatch repair. Curr Biol. 31. doi:10.1016/j.cub.2020.12.018. 10.1016/j.cub.2020.12.018 PMC8281387 33417883

[jkab205-B32] Rossi SE , Foiani M, Giannattasio M. 2018. Dna2 processes behind the fork long ssDNA flaps generated by Pif1 and replication-dependent strand displacement. Nat Commun. 9. doi:10.1038/s41467-018-07378-5. 10.1038/s41467-018-07378-5 PMC6240037 30446656

[jkab205-B33] Smith DJ , Whitehouse I. 2012. Intrinsic coupling of lagging-strand synthesis to chromatin assembly. Nature. 483: 434–438. doi:10.1038/nature10895. 22419157 10.1038/nature10895 PMC3490407

[jkab205-B34] Sparks MA , Burgers PM, Galletto R. 2020. Pif1, RPA, and FEN1 modulate the ability of DNA polymerase d to overcome protein barriers during DNA synthesis. J Biol Chem. 295. doi:10.1074/jbc.RA120.015699. 10.1074/jbc.RA120.015699 PMC7681027 32913126

[jkab205-B35] Stith CM , Sterling J, Resnick MA, Gordenin DA, Burgers PM. 2008. Flexibility of eukaryotic Okazaki fragment maturation through regulated strand displacement synthesis. J Biol Chem. 283. doi:10.1074/jbc.M806668200. 10.1074/jbc.M806668200 PMC2590699 18927077

[jkab205-B36] Stodola JL , Burgers PM. 2016. Resolving individual steps of Okazaki-fragment maturation at a millisecond timescale. Nat Struct Mol Biol. 23:402–408. doi:10.1038/nsmb.3207. 27065195 10.1038/nsmb.3207 PMC4857878

[jkab205-B37] Tubbs A , Sridharan S, van Wietmarschen N, Maman Y, Callen E, et al 2018. Dual roles of poly(dA:dT) tracts in replication initiation and fork collapse. Cell. 174: 1127–1142.e19. doi:10.1016/j.cell.2018.07.011. 30078706 10.1016/j.cell.2018.07.011 PMC6591735

[jkab205-B38] Yeung R , Smith DJ. 2020. Determinants of replication-fork pausing at tRNA genes in Saccharomyces cerevisiae. Genetics. 214: 825–838. doi:10.1534/GENETICS.120.303092. 32071194 10.1534/genetics.120.303092 PMC7153945

[jkab205-B39] Zhou ZX , Lujan SA, Burkholder AB, Garbacz MA, Kunkel TA. 2019. Roles for DNA polymerase δ in initiating and terminating leading strand DNA replication. Nat Commun. doi:10.1038/s41467-019-11995-z. 10.1038/s41467-019-11995-z PMC6728351 31488849

